# Development of flavivirus subviral particles with low cross-reactivity by mutations of a distinct antigenic domain

**DOI:** 10.1007/s00253-023-12817-5

**Published:** 2023-10-13

**Authors:** Koshiro Tabata, Yukari Itakura, Takuma Ariizumi, Manabu Igarashi, Hiroko Kobayashi, Kittiya Intaruck, Mai Kishimoto, Shintaro Kobayashi, William W. Hall, Michihito Sasaki, Hirofumi Sawa, Yasuko Orba

**Affiliations:** 1 Division of Molecular Pathobiology, International Institute for Zoonosis Control, Hokkaido University, Sapporo, Hokkaido 001-0020 Japan; 2 Institute for Vaccine Research and Development, Hokkaido University, Sapporo, 001-0021 Japan; 3 Division of Global Epidemiology, International Institute for Zoonosis Control, Hokkaido University, Sapporo, Hokkaido 001-0020 Japan; 4 International Collaboration Unit, International Institute for Zoonosis Control, Hokkaido University, Kita-Ku, Sapporo, N20, W10001-0020 Japan; 5 Laboratory of Veterinary Microbiology, Osaka Metropolitan University, Izumisano, 598-8531 Japan; 6 Laboratory of Public Health, Faculty of Veterinary Medicine, Hokkaido University, Sapporo, 060‑0818 Japan; 7 Global Virus Network, Baltimore, MD 21201 USA; 8 National Virus Reference Laboratory, University College Dublin, Belfield, Dublin, 4 Ireland; 9 One Health Research Center, Hokkaido University, Sapporo, Hokkaido 001-0020 Japan

**Keywords:** Mosquito-borne flavivirus, Insect-specific flavivirus, Subviral particle, Fusion loop domain, Cross-reactivity, Serodiagnosis

## Abstract

**Abstract:**

The most conserved fusion loop (FL) domain present in the flavivirus envelope protein has been reported as a dominant epitope for cross-reactive antibodies to mosquito-borne flaviviruses (MBFVs). As a result, establishing accurate serodiagnosis for MBFV infections has been difficult as anti-FL antibodies are induced by both natural infection and following vaccination. In this study, we modified the most conserved FL domain to overcome this cross-reactivity. We showed that the FL domain of lineage I insect-specific flavivirus (ISFV) has differences in antigenicity from those of MBFVs and lineage II ISFV and determined the key amino acid residues (G106, L107, or F108), which contribute to the antigenic difference. These mutations were subsequently introduced into subviral particles (SVPs) of dengue virus type 2 (DENV2), Zika virus (ZIKV), Japanese encephalitis virus (JEV), and West Nile virus (WNV). In indirect enzyme-linked immunosorbent assays (ELISAs), these SVP mutants when used as antigens reduced the binding of cross-reactive IgG and total Ig induced by infection of ZIKV, JEV, and WNV in mice and enabled the sensitive detection of virus-specific antibodies. Furthermore, immunization of ZIKV or JEV SVP mutants provoked the production of antibodies with lower cross-reactivity to heterologous MBFV antigens compared to immunization with the wild-type SVPs in mice. This study highlights the effectiveness of introducing mutations in the FL domain in MBFV SVPs with lineage I ISFV-derived amino acids to produce SVP antigens with low cross-reactivity and demonstrates an improvement in the accuracy of indirect ELISA-based serodiagnosis for MBFV infections.

**Key points:**

*• The FL domain of Lineage I ISFV has a different antigenicity from that of MBFVs.*

*• Mutated SVPs reduce the binding of cross-reactive antibodies in indirect ELISAs.*

*• Inoculation of mutated SVPs induces antibodies with low cross-reactivity.*

**Supplementary Information:**

The online version contains supplementary material available at 10.1007/s00253-023-12817-5.

## Introduction

Members of the Flaviviridae are enveloped, positive sense, single-stranded RNA viruses, which are mainly divided into mosquito-borne flaviviruses (MBFVs), tick-borne flaviviruses, insect-specific flaviviruses (ISFVs), and no-known-vector flaviviruses (Guarner and Hale 2019; Krol et al. 2019; Neufeldt et al. 2018; Pierson and Diamond 2020; Tabata et al. 2022). In recent years, countries where the MBFVs are known to be distributed have been extended because of a parallel expansion of the habitat of mosquitoes as a result of climate change, for example La Niña (Andersen and Davis 2017; Gould and Higgs 2009; Paz 2015; Semenza et al. 2022). In Southern Australia, where there had been no record of Japanese encephalitis virus (JEV) infection from a geographical or climatic point of view, JEV infection and a fatal case have been recently reported (Waller et al. 2022; Yakob et al. 2022). In France, *Aedes albopictus*, which was not originally found in that country, has been indigenous since 2010, and autochthonous cases of dengue transmitted by the *Aedes albopictus* have been reported (Marchand et al. 2013). Thus, MBFV infections may cause outbreaks in non-endemic areas and have become a significant concern for public health.

Although most infections with MBFVs are asymptomatic, JEV and West Nile (WNV) infections cause fatal encephalitis, and Zika virus (ZIKV) infection is related to microcephaly in newborns when pregnant women are infected (Brasil et al. 2016; Chancey et al. 2015; Misra and Kalita 2010). Dengue virus (DENV) infection results in classical dengue fever, including fever, muscle, and joint pain symptoms usually without serious illness in the initial infection (Vasilakis et al. 2011). Because there are four different serotypes (1 to 4) of DENV, pre-existing anti-DENV antibodies acquired during the initial infection can enhance a different serotype of DENV in secondary infection, causing severe illness and dengue hemorrhagic fever (Halstead 1988, 2003; Whitehead et al. 2007). This is termed antibody-dependent enhancement (ADE). Cohort studies have shown that antibodies induced not only by DENV infection but also by ZIKV infection and JEV vaccination cause ADE following DENV infection (Anderson et al. 2011; Katzelnick et al. 2017, 2020). To control these MBFV infections, there have been concerted efforts to develop specific therapies and vaccines. However, no specific medications have been approved to date, and the number of effective vaccines is limited (Barrows et al. 2016; Boldescu et al. 2017; Kok 2016; Stiasny et al. 2012).

It is important to perform epidemiological studies using serodiagnosis for our understanding of the distribution of MBFVs. There are several available serodiagnostic methods, including detection of virus-specific binding or measurement of neutralizing antibodies. While the neutralizing test is the most accurate and specific serological method for diagnosis, it is necessary to employ high-level biosafety level facilities for studies of highly pathogenic live viruses (Inagaki et al. 2016; Maezono et al. 2021). Therefore, enzyme-linked immunosorbent assay (ELISA)-based serodiagnostic methods for the detection of virus species-specific antibodies offer a useful alternative approach. However, antibodies induced by flavivirus infection have cross-reactivity to other flavivirus species, and as such, it is often difficult to detect virus species-specific antibodies by ELISA (Kuno 2003; Martin et al. 2000). Most of cross-reactive antibodies recognize the precursor membrane (prM) and envelope (E) proteins, which are highly conserved among MBFVs (Crill and Chang 2004; Dejnirattisai et al. 2010; Rathore and St John 2020). In particular, the fusion loop (FL) domain in the domain II of E protein (EDII) is known to be the most conserved in the flavivirus structural proteins (Chiou et al. 2008). The FL domain in EDII has been reported as containing the main epitopes for cross-reactive antibodies eliciting ADE (de Alwis et al. 2014; Gunawardana and Shaw 2018; Vogt et al. 2011). Domains I and III of E (EDI and EDIII) predominantly contain virus-specific epitopes (Chiou et al. 2008; Crill and Chang 2004; Roehrig et al. 1998; Stiasny et al. 2006; Sukupolvi-Petty et al. 2007). Therefore, in order to inhibit the binding of anti-FL domain antibodies, E proteins with artificial mutations in the FL domain have been reported to be useful to serve as antigens for establishment of serodiagnostic methods (Dai et al. 2021; Dejnirattisai et al. 2015; Rockstroh et al. 2017; Tsai et al. 2013). However, mutations in the FL domain resulted in the disruption of the original epitopes in virus particles and/or reduction of the protein expression (Dai et al. 2021; Slon-Campos et al. 2019; Urakami et al. 2017; Vogt et al. 2011). Thus, it is difficult to synthesize recombinant protein of MBFVs with both elimination of cross-reactive epitopes and retention of the original epitopes.

Recently, there are many reports of the isolation of new flavivirus species, especially ISFVs (Colmant et al. 2021; Orba et al. 2021; Piyasena et al. 2019; Wastika et al. 2020). The ISFVs are phylogenetically divided into lineages I (classical ISFV) and II (dual-host affiliated ISFV). Previous studies have shown that lineage II ISFVs are phylogenetically and antigenically close to MBFVs, while lineage I ISFVs are not (Auguste et al. 2021; Colmant et al. 2021; Guzman et al. 2018; Harrison et al. 2020; Hobson-Peters et al. 2019; Tabata et al. 2022; Pauvolid-Correa et al. 2015). The present study has aimed to create MBFV subviral particles (SVPs) with low cross-reactivity by replacing the amino acids from different antigenic ISFVs to maintain the epitopes in the SVP antigens and to develop indirect ELISA-based serodiagnostic systems using the mutated SVPs.

## Materials and methods

### Cell culture

Vero 9013 cells (Vero cells; JCRB, Ibaraki, Japan) were maintained in Dulbecco’s modified Eagle medium (DMEM, Nissui, Tokyo, Japan) supplemented with 10% fetal bovine serum (FBS) at 37 °C under 5% CO_2_. K562 cells were maintained in RPMI1640 (Nissui) supplemented with 10% FBS at 37 °C under 5% CO_2_. *Aedes albopictus-*derived C6/36 mosquito cells (ATCC, Manassas, VA, USA) were maintained in Eagle’s minimum essential medium (MEM, Nissui) supplemented with 10% FBS and nonessential amino acids at 28 °C under 5% CO_2_. Expi293F cells (Thermo Fisher Scientific, Waltham, MA, USA) were grown in Expi293 expression medium (Thermo Fisher Scientific) at 37 °C under 8% CO_2_.

### Viruses

The following viruses were used in this study: DENV type 2 (DENV2) hu/INDIA/09–74 (accession no. LC367234), ZIKV African strain MR766-NIID and Asian strain PRVABC59 (accession no. LC002520 and KU501215, respectively), JEV Beijing (accession no. L48961), WNV lineage I strain NY-99 and lineage II strain Zmq16m11 (accession no. KC407666.1 and LC318700.1, respectively), Psorophora flavivirus (PSFV, accession no. LC567151), Barkedji virus (BJV, accession no. LC497470), cell-fusing agent virus (CFAV, accession no. LC770212), and Culex flavivirus (CxFV, accession no. LC770211). All viruses were propagated in C6/36 cells in MEM containing 2% FBS. After 5 days, culture supernatants were collected and stored at − 80 °C until use.

### Alignment analysis

**Fig. 1:**
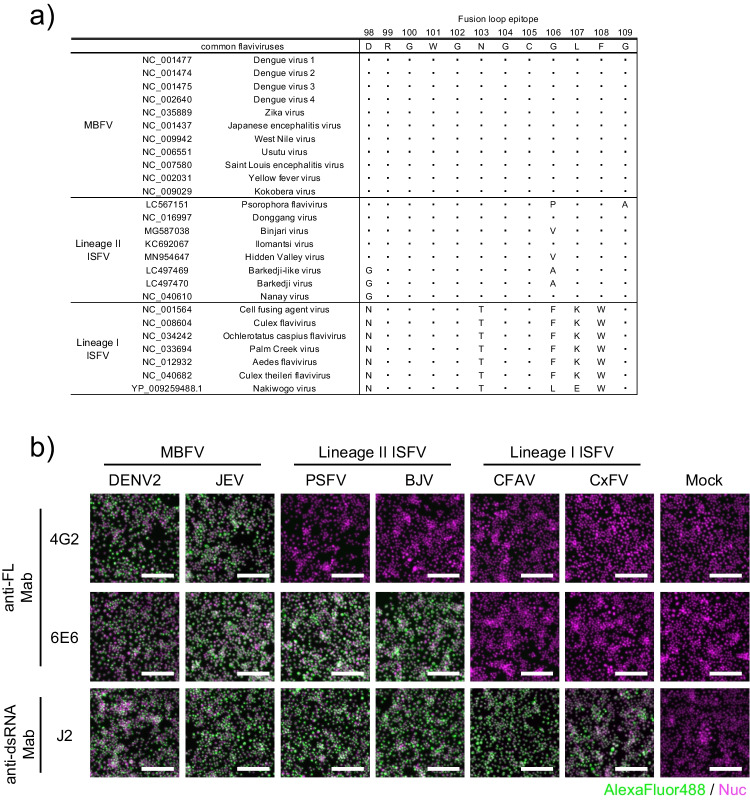
Comparison of the homology and antigenicity of the FL domains in flaviviruses. **a** Sequence alignment of the FL domains in pathogenic mosquito-borne flaviviruses (MBFVs), lineage I insect-specific flaviviruses (ISFVs), and lineage II ISFVs. **b** Antigenicity in the FL domain of MBFV, lineage II, and lineage I ISFVs was evaluated using anti-FL monoclonal antibodies (4G2 and 6E6). Viral replication was evaluated using anti-double-stranded RNA monoclonal antibody (J2) as control. DENV2, dengue virus type 2; JEV, Japanese encephalitis virus; PSFV, Psorophora flavivirus; BJV, Barkedji virus; CFAV, cell-fusing agent virus; CxFV, Culex flavivirus (CxFV); Nuc, nucleus. Scale bar indicates 100 µm

Amino acid sequences of various flaviviruses were obtained from the GenBank database. The accession number of each virus is described in Fig. 1A. The MUSCLE protocol was used to align the amino acid sequences using the CLC Genomics Workbench 20.1 (Qiagen, Hilden, Germany).

### Focus-forming assay (FFA)

Vero cells (1.5 × 10^5^ cells/well) in 24-well plates (Corning, Tewksbury, MA, USA) were inoculated with tenfold serial dilutions of each virus and incubated for 1 h at 37 °C (Vero cells) or 28 °C (C6/36 cells). An overlay medium was added (MEM containing both 0.5% methyl cellulose and 2% FBS) and incubated for 32 h (ZIKV, JEV, and WNV) or 72 h (DENV2). After incubation, cells were fixed with prechilled methanol at − 30 °C for more than 30 min. Fixed cells were blocked with phosphate-buffered saline (PBS) containing 1% bovine serum albumin (BSA) at room temperature (RT) for 30 min. Anti-flavivirus nonstructural protein 1 (NS1) 4G4 monoclonal antibody (mAb) (kindly provided by Prof. Roy Hall, University of Queensland, St Lucia, Queensland, Australia) in 0.1% BSA-PBS was then added to each well and incubated at RT for 1 h. The 4G4 mAb Alexa Fluor 488-conjugated anti-mouse immunoglobulin (Ig) G antibody (Invitrogen, Waltham, MA, USA) in 0.1% BSA-PBS was applied as a secondary antibody at RT for 1 h, and viral focus images were acquired using a fluorescence microscope (IX73, Olympus, Tokyo, Japan). Virus titers were calculated based on the number of foci and expressed as focus-forming units (ffu) per milliliter.

### Indirect fluorescence assay (IFA)

C6/36 cells were infected with DENV2, JEV, PSFV, or BJV at multiplicity of infection (MOI) of 1. CFAV and CxFV were 100-fold diluted and inoculated into C6/36 cells. Supernatants (100 µL) of infected cells with CFAV or CxFV were collected at 12 h post-infection (hpi) and 72 hpi for quantitative reverse transcription PCR (RT-qPCR). Following incubation for 4 days, cells were fixed with 4% paraformaldehyde (PFA) and blocked with PBS containing 1% BSA for 30 min at RT. Two anti-FL 4G2 (ATCC) and BJ-6E6 mAbs (6E6, kindly provided by Prof. Roy Hall, University of Queensland, St Lucia, Queensland, Australia) (Harrison et al. 2020) and an anti-double-stranded RNA (dsRNA) J2 Mab (Nordic MUbio, Susteren, Netherlands) were used as the primary antibodies with incubation at RT for 1 h. Alexa Fluor 488-conjugated anti-mouse IgG antibody (Invitrogen) in the presence of Hoechst 33342 was applied as a secondary antibody at RT for 1 h. Images were acquired using a fluorescence microscope (IX73). Experiments were independently conducted twice, and representative images are shown.

### Protein expression and purification of SVPs

The coding sequence for the precursor membrane and envelope protein (prME) from the DENV2 hu/INDIA/09–74 (1–661, accession no. LC770213), ZIKV MR766-NIID (1–672, accession no. LC770214), JEV Beijing (1–667, accession no. LC770215), and WNV NY-99 strains (1–668, accession no. LC770216) was optimized for human expression bias (GeneArt, Life Technologies) and cloned into the pCXSN plasmid (Sasaki et al. 2018) with a signal sequence derived from core protein sequences at the N-terminus (23 aa) of the JEV Beijing strain. SVPs were expressed in Expi293F cells following the manufacturer’s instructions. SVP mutants were created by inserting the following amino acid replacements, D98N, N103T, G106F/L, L107K/E, and F108W, in the FL domain. Culture supernatant fluids were harvested and clarified by centrifugation and filtration using a 0.4-µm filter. Thereafter, fluids were precipitated by ultracentrifugation with a 20% sucrose cushion at 153,720 × *g* for 2 h. Precipitated pellets were resuspended with PBS supplemented with 1 mM MgCl_2_ and 1 mM CaCl_2_ (PBS/MgCl_2_/CaCl_2_). SVPs were further purified *via* ultracentrifugation through a 10–50% sucrose gradient in PBS at 153,720 × *g* for 3 h. Each fraction was collected and analyzed by sodium dodecyl sulfate polyacrylamide gel electrophoresis (SDS-PAGE) with Coomassie brilliant blue (CBB) staining and sandwich ELISA (Fig. S1). E protein-enriched fractions were precipitated by ultracentrifugation with PBS at 153,720 × *g* for 2 h. The pellet was resuspended with PBS/MgCl_2_CaCl_2_. The amount of E protein in purified SVPs was assessed by SDS-PAGE followed by CBB staining using BSA as a standard.

### Immunoblotting

Immunoblotting was performed as previously described (Tabata et al. 2022). Briefly, supernatants (5 µL/well) or lysates (5 µL/well) of transfected cells and purified SVPs (200 ng of E protein/well) were separated using SDS-PAGE and transferred onto polyvinylidene difluoride (PVDF) membranes (Millipore, Burlington, MA, USA). Following blocking with 5% skim milk-PBS with 0.01% Tween20, the following primary antibodies were employed: for FL, anti-FL monoclonal antibody (clone 4G2); E, rabbit anti-DENV E polyclonal antibody (GeneTex, Irvine, CA, USA), rabbit anti-ZIKV E polyclonal antibody (GeneTex), rabbit anti-JEV E polyclonal antibody (GeneTex), and rabbit anti-WNV E polyclonal antibody (Novus Biologicals, CO, USA); prM, rabbit anti-DENV prM polyclonal antibody (GeneTex), rabbit anti-ZIKV prM polyclonal antibody (GeneTex), and rabbit anti-WNV prM polyclonal antibody (Abcam, Cambridge, UK). HRP-conjugated anti-mouse IgG antibody or rabbit IgG antibody (Thermo Fisher Scientific) was used as the secondary antibody with Immobilon Western HRP Substrate (Millipore). HRP-conjugated anti-β-actin polyclonal antibody (MBL, Tokyo, Japan) was used as control for cell lysate samples. The signal was visualized using an ImageQuant 800 (Cytiva, Marlborough, MA, USA). Experiments were independently conducted twice, and representative data are shown.

### Sandwich ELISAs

E protein mAb clone 402 (kindly provided by Kanonji Institute of the Research Foundation for Microbial Diseases of Osaka University, Kagawa, Japan) was coated in a 384-well plate (Corning) overnight at 4 °C. Non-specific binding was blocked with 1% BSA for 1 h at RT. Gradient fractions were added to each well and incubated for 2 h at 37 °C. After washing five times with PBS containing 0.01% Tween20, the plate was incubated for 1 h at 37 °C with HRP-conjugated anti-E Mab clone 402. After washing, 1-Step Ultra TMB-ELISA Substrate Solution (Thermo Fisher Scientific) was added, followed by incubation at RT for 5 min, and the reaction was stopped by the addition of 2 M sulfuric acid. The optical density (OD) value was measured at 450 nm using a GloMax Discover Microplate Reader (Promega, Madison, WI, USA). The OD value was evaluated in triplicate in two independent experiments and representative data are shown.

### Production of virus antibodies

Interferon-α/β and interferon-γ receptor-deficient AG129 mice were obtained from Marshall BioResources (Hull, East Yorkshire, UK), and bred in-house in our animal facility. For standard mouse-infected serum, 16–18-week-old male AG129 mice (*n* = 3) were inoculated subcutaneously with 1.0 × 10^5^ ffu of DENV2 hu/INDIA/09–74 or ZIKV MR766-NIID strains. Six-week-old female C3H (*n* = 3) or BALB/c mice (*n* = 3) (SLC, Shizuoka, Japan) were subcutaneously inoculated with 1.0 × 10^4^ ffu of JEV Beijing or of WNV NY-99 strains, respectively. For challenge mouse-infected serum, 10 or 15-week-old AG129 mice (*n* = 3, two 10-week-old male mice and a 15-week-old female mouse) or 6-week-old female BALB/c mice (*n* = 6) were inoculated subcutaneously with 1.0 × 10^3^, 1.0 × 10^4^, 1.0 × 10^5^, and 1.0 × 10^6^ ffu of ZIKV PRVABC5 or 1.0 × 10^4^ ffu of WNV Zmq16m11, respectively. The body weights (BWs) of the virus-inoculated mice were monitored daily. Mice were euthanized with isoflurane anesthesia when they showed a 10% decrease in their BW, and sera were then collected.

For mice immunizations, six-week-old female BALB/c mice were subcutaneously inoculated with SVP (1 µg of E protein content) admixed with an equivalent volume of Imject Alum Adjuvant (Thermo Fisher Scientific) twice at a 3-week interval. Three days after the second immunization, mice were euthanized using excess isoflurane anesthesia, and sera were collected. All infected and immunized sera were inactivated by incubation at 56 °C for 30 min and stored at − 80 °C until use.

### Indirect ELISAs

Each SVP protein (250 ng/mL of E protein) for antibody titer and standard serum (500 ng/mL) of E protein for challenge serum were coated on 384-well plates overnight at 4 °C. Non-specific binding was blocked with 10% horse serum (HS) in PBS for 1 h at RT. Mouse sera were diluted at 100-, 500-, 1000-, 5000-, and 10,000-fold with 0.1% HS in PBS and incubated for 2 h at 37 °C. After five times washing, the plate was incubated for 1 h at 37 °C with HRP-conjugated anti-mouse IgG (Sigma-Aldrich, Merck, St. Louis, MO, USA), IgM (Thermo Fisher Scientific), IgA antibody (Abcam, Cambridge, UK), and total Ig (Thermo Fisher Scientific). Following washing, the signals were detected as previously described in a sandwich ELISA. Positive-to-negative ratios (P/N ratios) were calculated as OD values of the serum specimens were divided into the average OD value of three uninfected control murine sera. Endpoint titers were determined as the reciprocal of the highest dilution over eightfold blank values. Experiments using representative samples were independently conducted twice to validate reproducibility, and data in biological replicate (*n* = 3–6) are shown.

### Focus reduction neutralization tests (FRNTs)

FRNTs were performed as previously described (Tabata et al. 2022). Vero cells (7.5 × 10^4^ cells/well) were seeded in 48-well plates (Corning) 24 h before infection. Serial tenfold diluted serum and 50 ffu of each virus were mixed and incubated for 1 h at 37 °C. Cells were then inoculated with the serum-virus complexes and incubated for 1 h at 37 °C, and then treated with an overlay media and incubated for a further 32 h. Fixation and primary staining were conducted as described for FFA. HRP-conjugated anti-mouse Ig antibody was used as the secondary antibody (Thermo Fisher Scientific). Virus foci were stained with TrueBlue Peroxidase Substrate (Sera Care Life Sciences Inc., Milford, MA, USA). Neutralizing antibody titers were defined as the reciprocal of the highest serum dilution showing a 50% reduction in the number of foci compared with that of the virus control (FRNT_50_) as previously reported (Slon Campos et al. 2017). Experiments using representative samples were independently conducted twice to validate reproducibility, and data in biological replicates (*n* = 4–6) are shown.

### ADE assay

The flow cytometry-based ADE assay was performed as previously described with slight modifications (Tabata et al. 2022). Briefly, serial two-fold dilutions of serum specimens were incubated with DENV, ZIKV, JEV, or WNV (MOI of 1) for 1 h at 37 °C, and 5.0 × 10^4^ K562 cells in RPMI containing 10% FBS were added to each well in round-bottom 96-well plates (Corning). At 48 hpi (JEV and WNV) or 72 hpi (DENV2 and ZIKV), cells were fixed with 4% PFA, and permeabilized with PBS containing 0.2% BSA and 0.05% saponin. Virus-infected cells were stained with the 4G2 antibody and Alexa Fluor 488-conjugated goat anti-mouse IgG antibody. Cells were counted using a FACSCanto (BD Biosciences, Franklin Lakes, NJ, USA) or LSRFortessa (BD Biosciences) and analyzed using FlowJo software version 10.7.1 (Tree Star Inc., Ashland, OR, USA). Data were expressed as ADE, calculating the area under the curve using GraphPad Prism software version 8.0 (GraphPad Software, San Diego, CA, USA). Experiments using representative samples were independently conducted twice to validate reproducibility, and data in the biological replicates (*n* = 4–6) are shown.

### Statistical analysis

All statistical analyses were performed using the GraphPad Prism software version 8.0. Statistical analysis is described in the figure legends for each experiment.

## Results

### Homology and antigenicity of the FL domain

The FL domain is highly conserved among flaviviruses, including MBFVs and ISFVs, and consequently, anti-FL domain antibodies exhibit broad cross-reactivity against most flaviviruses. To assess the homology of the FL domain in flaviviruses, we conducted alignment analysis using 12 amino acid sequences of FL domains among MBFVs and lineages I and II ISFVs (Fig. 1a). This revealed that lineage I ISFVs contain significantly more different amino acid residues in the FL domain compared with those in lineage II ISFVs and MBFVs (Fig. 1a).

We then performed IFA to evaluate the antigenicity of the FL domain using two different mAbs against the FL domains, clones 4G2 and 6E6. The 4G2 mAb was established by immunization of DENV antigen and is cross-reactive to the FL domains of MBFVs (Henchal et al. 1982). The 6E6 mAb was established by immunization of lineage II ISFV Binjari virus and had broader cross-reactivity compared with the FL domains of MBFVs and lineage II ISFVs when compared to the 4G2 mAb (Harrison et al. 2020). Previous study also reported that these mAbs recognize different epitopes (Harrison et al. 2020). Therefore, we used both 4G2 and 6E6 mAbs as representative anti-FL mAb which recognized different amino acids in the FL domains. Both 4G2 and 6E6 mAbs bound to DENV2- and JEV-infected cells, and viral antigen in cells infected with lineage II ISFVs (PSFV and BJV) were detected by mAb 6E6, but not by 4G2. However, neither of these mAbs bound to cells infected with lineage I ISVFs (CFAV and CxFV). Viral replication of these MBFVs, lineage I, and lineage II ISFVs was confirmed by staining for viral dsRNAs using mAb J2 (Fig. 1b). Additionally, RT-qPCR verified an increase in levels of viral RNA of CFAV and CxFV (Fig. S2), demonstrating that both of the anti-FL mAbs (4G2 and 6E6) did not bind with cells infected with CFAV and CxFV which belong to lineage I ISFVs. Previous study has also shown that both 4G2 and 6E6 mAbs were not reactive to other lineage I ISFVs (Harrison et al. 2020). Thus, the FL domain of lineage I ISFVs seems to have different antigenicity compared with those of MBFVs and lineage II ISFVs.

### Mutations in the FL domain which prevent binding with anti-FL mAbs

**Fig. 2:**
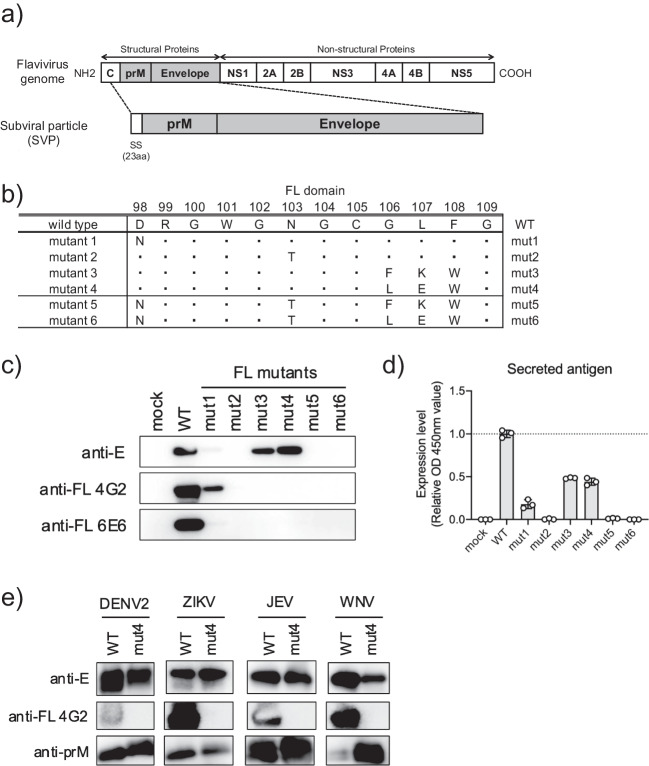
Subviral particle (SVP) mutants replacing the FL domain of lineage I ISFV. **a** Schematic representation of the SVP constructs. C, capsid; prM, precursor membrane; NS, nonstructural protein; SS, signal sequence. **b** FL domains of generated SVP mutants based on JEV SVPs. **c** Immunoblotting of supernatant transfected with plasmids encoding each SVP. Anti-JEV envelope (E) polyclonal antibody was used as anti-E antibody, and 4G2 and 6E6 monoclonal antibodies were used as anti-FL antibodies. **d** A sandwich enzyme-linked immunosorbent assay (ELISA) was performed using supernatants of transfected cells. Expression levels of SVPs were expressed as relative OD values to SVP WT. **e** Immunoblotting of purified SVP proteins for DENV2, Zika virus (ZIKV), JEV, and West Nile virus (WNV). Virus-specific anti-E and anti-prM polyclonal antibodies and anti-FL 4G2 mAb were employed

To determine the amino acid residues responsible for the binding of the anti-FL mAbs, we conducted immunoblotting using the supernatants of Expi293F cells transfected with the plasmid encoding JEV SVP. The JEV SVP wild type (WT) was composed of prME proteins containing the capsid (C)-derived 23 amino acids at the C-terminus as a signal peptide (Fig. 2a). SVP mutants (mut1-6) were created by replacing the amino acid residues of the FL domain of the E protein in JEV with those of lineage I ISFVs (Fig. 2b).

Immunoblotting using anti-E antibody demonstrated that JEV SVP WT, mut1 (D98N), mut3 (G106F, L107K, and F108W), and mut4 (G106L, L107E, and F108W) were secreted in the culture supernatant of the SVP encoding plasmids transfected Expi293F cells (Fig. 2c). In contrast, three SVP mutants (mut2, mut5, and mut6) containing N103T were not recognized in supernatants from the cells (Fig. 2c) and only faintly detected in the cell lysates (Fig. S3). The SVP mut3 and mut4 impaired the binding of anti-FL 4G2 and 6E6 mAbs, although mut1 was still detectable by the anti-FL 4G2 mAb (Fig. 2c). We also conducted a sandwich ELISA to evaluate the protein expression level and particle formation efficiency. The sandwich ELISA using anti-E mAb (clone 402) showed greater levels of SVP mut3 and mut4 compared to the SVP mut1 in supernatants of cells transfected with plasmids encoding SVPs (Fig. 2d). We consequently focused on the SVP mut4 for further experiments as SVP mut4 had comparable protein expression levels to SVPmut3 on sandwich ELISA, and immunoblotting showed higher expression levels of SVP mut4 compared to that of SVP mut3.

In addition to JEV, SVP WT and mut4 with G106L, L107E, and F108W mutations were generated for DENV2, ZIKV, and WNV. Each SVP was purified *via* ultracentrifugation and was assessed for binding with anti-FL antibody. Immunoblotting showed that both SVP WT and mut4 reacted with virus-specific anti-E and anti-prM antibodies, whereas anti-FL 4G2 mAb only bound to SVP WT but not to SVP mut4 (Fig. 2e).

### Identification of virus-specific antibody subclasses in MBFV-infected serum by indirect ELISAs using SVP mut4

By replacing amino acid residues of the FL domain of MBFV SVP with those of the lineage I ISFV (G106L, L107E, and F108W), SVP mut4 did not bind anti-FL antibody. To evaluate the binding activities of flavivirus-infected sera with the SVPs, indirect ELISAs were performed using SVP WT or mut4 as antigens, where the amounts of SVPs were adjusted with the amount of E protein. Flavivirus-infected mouse serum (standard serum) was produced by infection with DENV2 (hu/INDIA/09–74), ZIKV (MR766-NIID, African), JEV (Beijing), and WNV (NY-99, lineage I), which are the same strains as the SVP antigen. Indirect ELISA was performed to measure signals of positive-to-negative ratio (P/N ratio) of IgM, IgG, IgA, and total Ig, which bind with each SVP antigen in the flavivirus-infected sera to evaluate the antibody subclass with high virus species-specific signals.

**Fig. 3:**
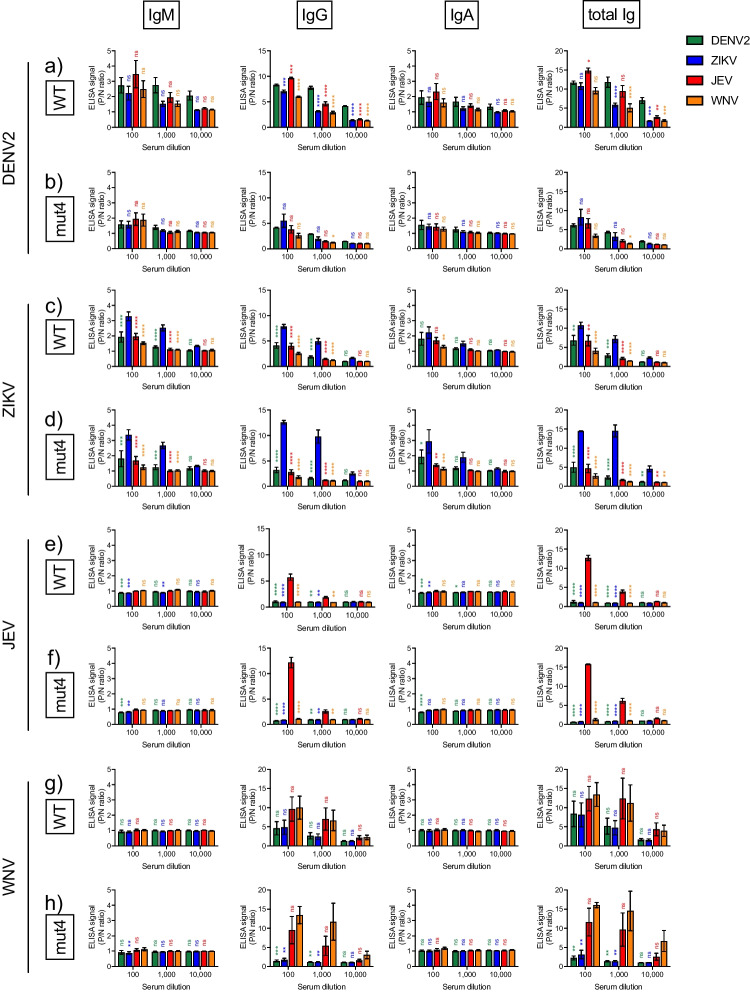
Indirect ELISAs with SVP WT and mut4 using flavivirus-infected sera with same viral strain as SVP (standard serum). (a–h) Immunoglobulin (Ig) M, IgG, IgA, and total IgG to each SVP antigen in mouse sera infected with DENV2 (a and b), ZIKV (c and d), JEV (e and f), or WNV (g and h) were measured by indirect ELISA. SVP WT (a, c, e, and g) and SVP mut4 (b, d, f, and h) were used as indirect ELISA antigens. Values in the graphs are expressed as the mean ± sem of serum samples (*n* = 3). Statistical analysis was performed by two-way ANOVA with Dunnett’s multiple comparison test. All statistical significances of the differences are indicated in comparison to either DENV2 SVP (a and b), ZIKV SVP (c and d), JEV SVP (e and f), or WNV SVP (g and h) (*p < 0.01, **p < 0.005, ***p < 0.001, ****p < 0.0005)

It was difficult to determine DENV2-specific responses among IgM, IgG, IgA, and total Ig, because DENV2-infected sera broadly cross-reacted to heterologous viral antigens, even when the SVP mut4 was used (Fig. 3a and b). The ZIKV SVP WT antigen showed significantly higher signals of IgM, IgG, and total Ig in ZIKV-infected serum than those of other SVPs (Fig. 3c). The ZIKV SVP mut4 reacted with ZIKV-infected sera with significantly higher signals of all Ig subclasses compared with those of other mut4 SVPs (Fig. 3d). JEV-infected sera showed a JEV-specific response with low cross-reactivity to both of SVP WT and mut4 (Fig. 3e and f). In contrast to DENV2 or ZIKV-infected sera, no JEV-specific IgM or IgA responses were detected, whereas signals for IgG and total Ig to JEV WT and mut4 SVPs were significantly higher than other SVPs. In sera infected with WNV which belongs to the same serocomplex as JEV, the highest signals of IgG and total Ig to WNV mut4 SVP at all dilutions were observed, but the signals to WNV WT SVP were present at only 100-dilution (Fig. 3g and h). Similar to the binding properties of JEV-infected sera, specific antibody responses in the IgM and IgA subclasses were not observed in WNV-infected sera. In summary, virus-specific IgM, IgG, IgA, and total Ig responses were induced in ZIKV-infected sera, whereas viral specific IgG and total Ig responses were present in JEV- and WNV-infected sera. These observations indicate that the IgG and total Ig to each MBFV SVP are suitable for the detection of virus species-specific antibodies.

**Fig. 4:**
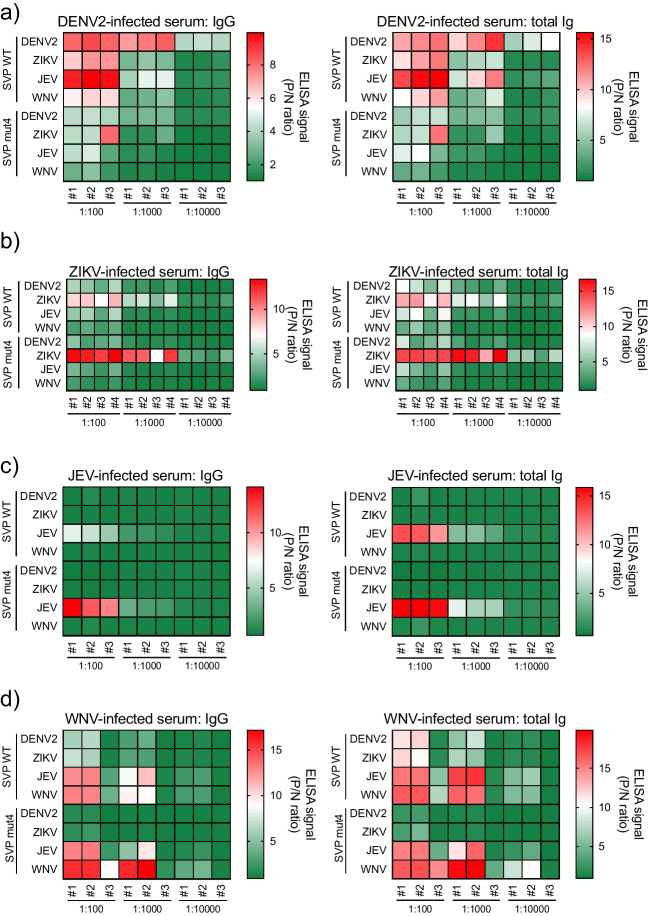
Heatmaps for cross-reactivities of IgG and total Ig in individual flavivirus-infected serum to each SVP WT and mut4 antigen (standard serum). Heatmaps of IgG and total Ig from individual mouse serum infected with DENV2 (**a**), ZIKV (**b**), JEV (**c**), and WNV (**d**). #, each individual mouse. The ELISA signals were expressed from green (low) to red (high)

Next, the IgG and total Ig signals in the MBFV-infected serum of individual mouse to each MBFV SVP antigen were analyzed. Heatmapping of the signals of DENV2-infected individual serum showed that the non-specific or cross-reactive IgG and total Ig responses to ZIKV or JEV SVP antigens had the highest signal at a 100-fold serum dilution, and after diluting each serum 1000-fold, the signal of IgG and total Ig to DENV2 SVP WT became higher than those of other SVP antigens (Fig. 4a). In ZIKV-infected individual sera, the highest signals of IgG and total Ig were observed with ZIKV SVP antigen (Fig. 4b). Moreover, cross-reactive signals to DENV2, JEV, and WNV SVP antigens were markedly decreased in SVP mut4 in all diluted serum. JEV-infected individual serum clearly showed specific binding to JEV SVP antigens without cross-reactive binding to DENV2, ZIKV, or WNV SVP WT or mut4 (Fig. 4c). Although similar signals in WNV SVP WT and JEV SVP WT antigens were observed in WNV-infected individual mouse serum, the IgG and total Ig responses to WNV SVP mut4 antigen were higher than that of JEV SVP mut4. The cross-reactive signals of WNV-infected serum to JEV SVP mut4 antigen were decreased by dilution, resulting in clear detection of virus-specific IgG and total Ig responses (Fig. 4d). These results indicate that the SVP mut4 when used as an antigen in the indirect ELISA system improves the detection of virus species-specific binding by decreasing cross-reactivity of antibodies from mice infected with MBFVs.

### Indirect ELISAs with SVP mut4 were applied to serodiagnosis using murine serum infected with different MBFV strains from different SVP antigens

SVP mut4 used as an antigen in the indirect ELISA detected virus-specific IgG and total Ig in the same virus species and strain of MBFV-infected serum (standard serum) as that of the SVPs, suggesting that the SVP mut4 can be employed as effective antigen in the indirect ELISA system for serodiagnosis of MBFV infections. Indirect ELISA with SVP mut4 was then performed using the sera of mice infected with different strains of ZIKV (PRVABC59, Asian) or WNV (Zmq16m11, lineage 2) (challenge serum) from the SVP antigen (strains of ZIKV MR766-NIID, Africa or WNV NY99, lineage 1) to measure the IgG responses in the infected serum.

**Fig. 5:**
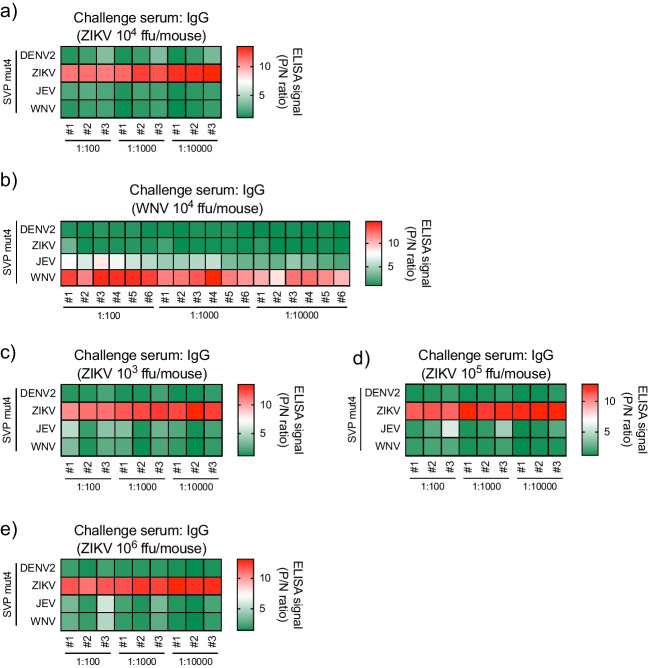
Heatmaps for cross-reactivity of IgG in serum from infected mouse with different strains of ZIKV (PRVABC59) and WNV (Zmq16m11) to each SVP mut4 antigen (challenge serum). **a**, **b** Heatmaps of IgG from individual mouse serum infected with ZIKV (**a**) and WNV (**b**) at 10^4^ ffu/mouse. **c**–**e** ZIKV-infected mouse serum was produced by inoculation with 10^3^, 10^5^, and 10^6^ ffu/mouse, respectively. Heatmap for IgG from individual mouse serum infected with ZIKV at 10^3^ (**c**), 10^5^ (**d**), and 10.^6^ (**e**) ffu/mouse. The ELISA signals were expressed from green (low) to red (high)

The indirect ELISA with SVP mut4 showed that IgG signal to ZIKV SVP mut4 was the highest even in individual challenge serum to ZIKV, but the signals to DENV2, JEV, and WNV SVP mut4 were markedly lower (Fig. 5a). In the WNV-infected challenge serum, IgG signals to WNV SVP mut4 were highest in all individuals, and cross-reactive IgG signal to JEV SVP mut4 was relatively suppressed at all dilutions of the challenge serum (Fig. 5b).

We then examined the virus infectious dose on the indirect ELISA P/N ratio of challenge serum. ZIKV-infected challenge sera were produced by inoculation of different amounts of ZIKV (10^3^, 10^5^, or 10^6^ ffu) into individual mice. The indirect ELISA with ZIKV SVP mut4 demonstrated that the highest IgG signal to ZIKV SVP mut4 was observed in all challenge serum with different infectious doses, but low cross-reactivity of IgG responses to other MBFV SVP mut4 was observed (Fig. 5c–e). Thus, the indirect ELISA system with the SVP mut4 allows the determination of the species of MBFV by recognition of virus species-specific IgG antibodies even when the infected viral strain was mismatched to the strain of SVP.

### Induction of virus-specific antibodies by immunization with the SVP mut4

**Fig. 6:**
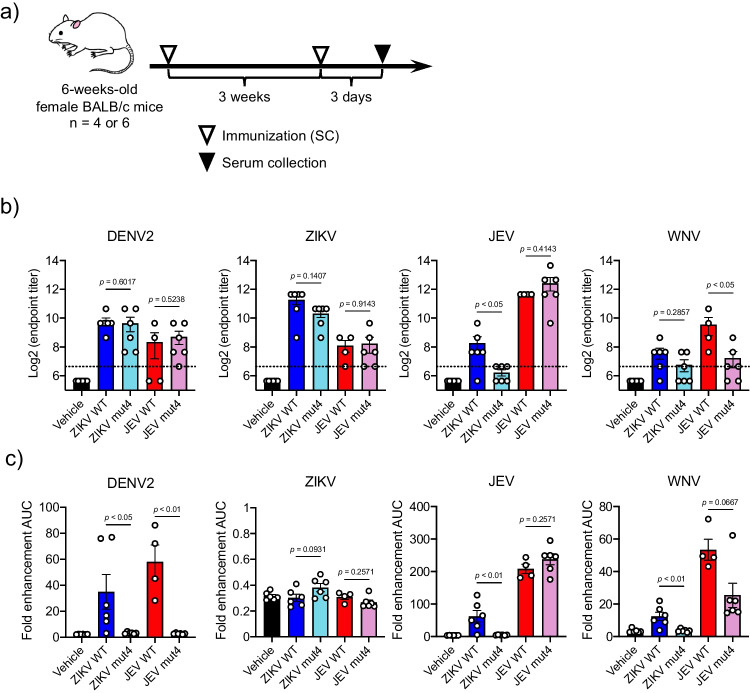
Binding, antibody-dependent enhancement (ADE), and neutralization activities of antibodies induced by immunization with the SVP mut4. **a** Schedule of immunization of mice. **b** Binding activities of each immunized sera were measured *via* indirect ELISAs using SVP WT antigen. **c** Each serum was evaluated for their enhancement of MBFV infections in K562 cells by flow cytometry. ADE activity was determined for the area under the curve shown in Fig. S4. Values in the graphs are expressed as the mean ± sem of serum samples (*n* = 6; vehicle, ZIKV WT, ZIKV mut4, and JEV mut4, *n* = 4; JEV WT). Dotted lines indicate detection limits. Statistical analysis was performed by Mann–Whitney *U* test

The substitution of amino acids in the FL domains of MBFVs with those of lineage I ISFV reduced the cross-reactive binding of MBFV-infected mouse serum to other MBFV SVPs. To evaluate whether the substitution of the FL domain of flavivirus SVPs could elicit virus-specific antibodies with low cross-reactivity, mice were subcutaneously immunized with a combination of SVP and alum adjuvant two times at 3-week intervals (Fig. 6a). The SVP WT and mut4 of ZIKV or JEV were used as immunogens because of their higher protein yield than those of DENV2 and WNV.

Indirect ELISAs with SVP WT antigens were then conducted to measure antibody titers in sera of the immunized mice (Fig. 6b). The endpoint titers of ZIKV or JEV SVP WT- and mut4-immunized sera against DENV2 SVP WT antigen showed no significant difference (*p* = 0.6017 and 0.5238, respectively). Similarly, the endpoint titers against ZIKV SVP WT antigen showed no significant difference between ZIKV or JEV SVP WT- and mut4-immunized sera (*p* = 0.1407 and 0.9143, respectively). The endpoint titers against JEV SVP WT antigen were significantly lower in sera immunized with ZIKV SVP mut4 compared to those immunized with the SVP WT (*p* < 0.05), whereas the JEV SVP immunization did not exhibit any difference in the titers between SVP WT and mut4 (*p* = 0.4143). The titers against WNV SVP WT antigen tended to decrease, but not significantly in sera immunized with ZIKV SVP mut4 compared to sera immunized with the SVP WT (*p* = 0.2857). In contrast, titers in sera immunized with JEV SVP mut4 were significantly decreased in comparison with those with the SVP WT (*p* < 0.05).

Antibodies induced by immunization with ZIKV or JEV SVPs were assessed for ADE activities (Figs. 6c and S4). ADE activities against infections of DENV2, JEV, and WNV but not ZIKV were significantly inhibited in the sera of mice immunized with ZIKV SVP mut4 compared to those with the SVP WT. ADE activity of JEV SVP mut4-immunized sera to DENV2 infection was also significantly inhibited compared to that of the SVP WT-immunized sera (*p* < 0.01). ADE activity against JEV infection was present in both JEV SVP WT- and mut4-immunized sera, whereas the activity against WNV, which belongs to the same serocomplex as JEV, was relatively lower in the SVP mut4-immunized sera compared to the SVP WT -immunized sera (*p* = 0.0667).

Neutralizing activity was also evaluated to confirm that the neutralizing epitope was not destroyed by the mutations in the FL domain. PRNT assays showed neutralization activity against ZIKV or JEV in a subset of mouse sera immunized with SVPs of ZIKV and JEV, and some immune sera from mice immunized with ZIKV SVP mut4 exhibited neutralizing activity against ZIKV infection comparable with those immunized with the SVP WT (*p* > 0.9999, positive rate of WT: 2/6 and positive rate of mut4: 2/6) (Fig. S5a). Sera from mice immunized with JEV SVP inhibited JEV infection regardless of whether SVP WT or mut4 was used as the immunogen (*p* = 0.9714, positive rate of WT: 2/4 and positive rate of mut4: 4/6) (Fig. S5b).

## Discussion

Antibodies to the FL domain exhibit cross-reactivities among the MBFVs as this is the most conserved region in the diverse and numerous species of flaviviruses. In the present study, we have demonstrated antigenic differences in the FL domain among MBFVs and ISFVs using anti-FL 4G2 and 6E6 mAbs and have determined key amino acid residues (G106, L107, or F108) involved in the recognition of the mAbs and the respective immune responses. Previous studies also demonstrated that single or double mutations in the G106 or/and K107 residues in FL domain of MBFV virus-like particles (VLPs) inhibited the bindings of major cross-reactive mAbs and double-mutated JEV VLPs (G106K and L107D) used as antigens in ELISAs provided an accurate determination of current JEV infections in human infections (Chao et al. 2019; Chiou et al. 2008; Crill and Chang 2004). In the present study, in addition to G106 and L107, a mutation was also introduced in F108, with reference to existing flavivirus structures. The SVP mut4 with G106L, L107E, and F108W in the FL domain was used in indirect ELISA antigen to sensitively detect binding of viral species-specific antibodies by suppressing cross-reactive bindings in the serum of mice infected with MBFVs compared to SVP WT (Fig. 4). The indirect ELISA system with SVP mut4 clearly discriminated ZIKV- or WNV-specific IgG antibodies from other MBFV-specific antibodies even when a viral strain of SVP antigen was different from infected viral strain (Fig. 5). Moreover, the serum of mouse immunized with ZIKV or JEV SVP mut4 showed relatively or significantly lower cross-reactivity to WNV antigen compared to that with SVP WT, respectively (Fig. 6b). Although there were no significant differences in cross-reactivity to DENV antigen between SVP mut4- and WT-immunized sera, the ADE activities of SVP mut4-immunized serum to DENV2 and WNV infections were significantly lower than these of SVP WT-immunized serum (Fig. 6c). Furthermore, SVP mut4 immunization showed a similar induction level of neutralizing antibodies to SVP WT immunization (Fig. S5). These results suggest that substitution of FL domain found in lineage I ISFV with that of MBFV SVP did not affect the original epitopes but change the sensitivity of cross-reactive antibodies. Thus, this mutation approach is useful for the production of SVP antigens with low cross-reactivity allowing the development of accurate serodiagnostic methods.

Interestingly, we observed that ZIKV-, JEV-, and WNV-infected serum presented higher virus-specific ELISA signals to mutated SVP antigens than to SVP WT antigens (Figs. 3 and 4). Flaviviruses undergo conformational changes through processing from immature to mature virions (Rey et al. 2017). Mature or immature virions are defined by the presence of prM protein. Immunoblotting of purified SVPs showed the SVPs contain prM proteins, suggesting that the SVP particles could be considered to be a partially immature form (Fig. 2e). Previous structural studies have reported that the prME protein in the immature flavivirus particle forms a spike appearance, and the FL domain is near the tip of the spike (Newton et al. 2021; Rey et al. 2018). Based on this structural information, it is expected that anti-FL antibodies may interfere with the bindings of antibodies to other epitopes on the E protein. Thus, it is speculated that virus-specific antibodies can access other epitopes on the mutated SVPs other than the FL domain, with the result that the ELISA signals were increased compared to when SVP WT is used as the antigen.

WNV-infected sera were reacted with JEV SVP even when using mutated SVP as well as WNV SVP (Figs. 3h and 4d). However, JEV-infected serum could specifically bind to JEV SVP antigen (Fig. 3e and f). These results suggested WNV and JEV infections to mice exhibit different profiles of antibody responses. In DENV2-infected serum, the mutated SVP did not clearly distinguish the DENV2-specific antibodies because the infected serum was strongly cross-reactive to heterologous viral antigens at high concentrations (Fig. 4a). A previous study showed that more than 80% of anti-DENV E mAbs isolated from DENV-infected patients cross-react with antigens in the DENV group and JEV (Dejnirattisai et al. 2010). These suggest that the prME protein of DENV2 has more cross-reactive epitopes than those of other MBFVs. To inhibit the binding of cross-reactive antibodies in WNV- and DENV2-infected serum, further studies are needed to reveal profiles of antibody responses by flavivirus infections and to determine other cross-reactive epitopes. In the present study, murine serum specimens were applied to validate the ELISA-based serodiagnosis, but further validation of the serodiagnosis using human specimens is necessary to establish an accurate serodiagnostic application.

Previous studies demonstrated that ZIKV- and WNV-infected sera can induce broadly cross-reactive antibodies, and the antibodies elicited ADE to other MBFV infections in mouse, non-human primates, and humans (Bardina et al. 2017; Katzelnick et al. 2017, 2020; Pantoja et al. 2017). Similarly, our results showed that ZIKV- and WNV-infected murine sera were cross-reactive with other MBFV SVP WTs, while mutation of the FL domain in the SVP reduced the binding of cross-reactive antibodies in ZIKV- and WNV-infected murine serum compared to SVP WT (Fig. 4b and d). This suggests that anti-FL antibodies may be frequently induced by MBFV infections. Furthermore, anti-FL mAbs showed cross-reactivities and non- or weak neutralizing activities to several flavivirus species, thereby inducing ADE (Berneck et al. 2020; Hurtado-Monzon et al. 2020). Therefore, in order to develop an effective vaccine against MBFV infections, epitopes for cross-reactive and ADE-prone antibodies, such as in the FL domain, need to be removed from vaccine antigen candidates.

In the present study, we found that immunization with the SVP mutated in the FL domain induced neutralizing antibodies without ADE activities (Figs. 6c and S5). This suggests that mutations in the FL domain may provide a promising approach to inhibit induction of ADE-prone antibodies and induce neutralizing antibodies. Supportively, a previous study demonstrated that vaccination of adenovirus vectors carrying ZIKV membrane (M) and E protein with the FL domain of lineage I ISFV elicited neutralizing antibodies that do not cause ADE and also protected from lethal virus infection (Dai et al. 2021). In their study, Dai and colleagues mutated the FL domain in the vaccine antigen and also removed the prM protein, which is known as cross-reactive epitope (Dejnirattisai et al. 2010). However, their results showed that the ZIKV antigens without the lineage I ISFV-derived FL domain induced cross-reactive ADE-prone antibodies even when the antigen contained only the M and E proteins. Hence, these findings suggest that flavivirus structural proteins carrying the FL domain of the lineage I ISFV may be useful as vaccine antigens against several MBFV infections. In order to develop an effective SVP-based vaccine against MBFV, we must determine how much and how often SVP antigen should be used for vaccination for the induction of effective neutralizing antibodies. These studies are currently underway.

### Supplementary Information

Below is the link to the electronic supplementary material.

Supplementary file1 (DOCX 1.91 MB)

## Data Availability

The datasets generated and analyzed during the current study are available from the corresponding author.
