# Supplementary material for: Development of flavivirus subviral particles with low cross-reactivity by mutations of a distinct antigenic domain
Source: Appl Microbiol Biotechnol. 2023 Oct 13;107(24):7515–29. doi: 10.1007/s00253-023-12817-5 (PMC10656323; doi:10.1007/s00253-023-12817-5)
Supplement: Supplementary file 1 — Supplementary file1 (DOCX 1.91 MB) [file 253_2023_12817_MOESM1_ESM.docx]

**Development of flavivirus subviral particles with low cross-reactivity by mutations of a distinct antigenic domain**

Koshiro Tabata^1,2^, Yukari Itakura^1,2^, Takuma Ariizumi^1^, Manabu Igarashi^4,5^, Hiroko Kobayashi^1^, Kittiya Intaruck^1^, Mai Kishimoto^1,3^, Shintaro Kobayashi^6^, William W. Hall^5,7,8^, Michihito Sasaki^1,2^, Hirofumi Sawa^2,5,7,9^, Yasuko Orba^1,2,5,*^

**Author affiliation:**

^1^ Division of Molecular Pathobiology, International Institute for Zoonosis Control, Hokkaido University, Sapporo, Hokkaido, 001-0020, Japan

^2^ Institute for Vaccine Research and Development, Hokkaido University, Sapporo, 001-0021, Japan

^3^ Laboratory of Veterinary Microbiology, Osaka Metropolitan University, Izumisano, 598-8531, Japan

^4^ Division of Global Epidemiology, International Institute for Zoonosis Control, Hokkaido University, Sapporo, Hokkaido, 001-0020, Japan

^5^ International Collaboration Unit, International Institute for Zoonosis Control, Hokkaido University, Sapporo 001-0020, Japan

^6^ Laboratory of Public Health, Faculty of Veterinary Medicine, Hokkaido University, 060‑0818, Japan.

^7^ Global Virus Network, Baltimore, Maryland, 21201, USA

^8^ National Virus Reference Laboratory, University College Dublin, Belfield, Dublin, 4, Ireland

^9^ One Health Research Center, Hokkaido University, Sapporo, Hokkaido, 001-0020, Japan

**^*^Corresponding author**:

E-mail: [orbay@czc.hokudai.ac.jp](mailto:orbay@czc.hokudai.ac.jp)

Postal address: International Institute for Zoonosis Control, Hokkaido University, N20, W10, Kita-ku, Sapporo, 001-0020, Hokkaido, Japan

**Supplementary Material and Method**

**Quantitative reverse transcription PCR (RT-qPCR)**

Aliquots of supernatants of Cell fusing agent virus (CFAV)- and Culex flavivirus (CxFV)-infected C6/36 cells were extracted using Direct-Zol kit (Zymo research, CA, USA). The amount of viral RNA was quantified using a PrimeScript One-step RT-PCR kit Ver.2 (Takara, Shiga, Japan) and each virus-specific primer set (for CFAV, primer 1: 5ʹ-AGA AAG CTC ACC AAC CAA CG-3ʹ, primer 2: 5ʹ-AGG GGT GTC AAC CGA AAA TG-3ʹ; for CxFV, primer 3: 5-ʹ-AGT TAT ATC AGA TGC CGA CGA C-3ʹ, primer 4: 5ʹ-AGA GCC CAC AAC ACT TCC GT-3ʹ), under the following thermal conditions: 42°C for 5 min, 95°C for 10 sec, and 40 cycles of 95°C for 5 sec and 60°C for 30 sec. Standard curves were generated using a series of diluted PCR products, and the RNA copy number per sample was calculated.

**Results**

Fig. S1 (a) SDS-PAGE analysis of Japanese encephalitis virus (JEV) subviral particles (SVPs) wild type (WT) using fractions of ultracentrifugation with 10-50% sucrose gradients. (b) Antigen capture enzyme-linked immunosorbent assay (ELISA) with gradient fractions of JEV SVP. (c to e) SDS-PAGE analysis of Dengue virus type 2 (DENV2) (c), Zika virus (ZIKV) (d) and West Nile virus (WNV) (e) SVP WT using fractions of ultracentrifugation with 10-50% sucrose gradients.

Fig. S2 Propagation of CFAV and CxFV were measured by RT-qPCR.

Fig. S3 Immunoblotting of cell lysate of transfected with plasmids encoded WT or mutant of JEV SVP. E: envelope.

Fig. S4 Fold enhancements of infected K562 cells with DENV2, ZIKV, JEV and WNV were measured by flow cytometry.

Fig. S5 Neutralization activities of each serum were measured by 50% focus reduction neutralization test (FRNT50). Values in the graphs are expressed as the mean ± sem of serum samples (n = 6; vehicle, ZIKV WT, ZIKV mut4, and JEV mut4, n = 4; JEV WT). Dotted lines indicate detection limits. Statistical analysis was performed by Mann–Whitney U test.
